# Fire needle therapy for blood stasis syndrome of plaque psoriasis: A protocol for systematic review and meta-analysis

**DOI:** 10.1097/MD.0000000000025312

**Published:** 2021-04-02

**Authors:** Gang Huang, Juan Yan, Jiahua Zou, Chuxiang Hu, Dongbei Huang, Qiang Huang, Peize Chen, Feiyan Zhang, Liping Gong

**Affiliations:** a Affifiliated Hospital of Jiangxi University of Traditional Chinese Medicine; b Jiangxi University of Traditional Chinese Medicine, Nanchang; c The First Affiliated Hospital of Gannan Medical College, Ganzhou, China.

**Keywords:** blood stasis syndrome, fire needle, plaque psoriasis, protocol, systematic review

## Abstract

**Background:**

Plaque psoriasis (PSO) is a common clinical chronic inflammatory skin disease. The incidence rate is increasing year by year due to the fast pace of work and unhealthy diet. Fire needle has been widely used in the treatment of PSO. However, the efficacy of fire needle for PSO is uncertain. Thus, the purpose of this systematic review is to evaluate the effectiveness and safety of fire needle for PSO (blood stasis syndrome).

**Methods:**

The following electronic databases will be searched from inception to October 2020:PubMed, Web of Science, Embase, Cochrane Library, China National Knowledge Infrastructure, WangFang Database, Chinese Science Journal Database, Chinese Biomedical Literature Database. In addition, other documents that meet the requirements will be manually searched, including conference papers, dissertations, etc. All randomized controlled trials using fire needle to treat PSO (blood stasis syndrome) that meet the criteria for inclusion will be included. The primary outcomes are clinical efficacy, Psoriasis area and severity index. Secondary outcomes include Itchy, TCM evaluation standard syndrome score, Dermatological quality of life index, and adverse events. To complete data synthesis and assess the risk of bias, we will use the RevMan V.5.3 software.

**Results:**

The review results will be published in a peer-reviewed journal.

**Conclusion:**

This study will provide high-quality evidence based medicine to evaluate the effectiveness and safety of fire needle for PSO (blood stasis syndrome), and further seek its scientific and effective chinese medicine treatment methods.

**INPLASY registration number:**

INPLASY202120007.

## 1 Introduction

Psoriasis is a chronic, immune-mediated polygenetic hereditary skin disease, with a global prevalence of children ranges from 0% to 1.37%, and from 0.51% to 11.43% in adults.^[1]^ According to statistics, PSO affects about 3.2% of the United States’ population,^[2,3]^ with a higher prevalence compared to China (0.47%).^[4]^ PSO, accounting for about 85% to 90%, is the most common subtype of psoriasis. Its typical features are mainly scaly erythema or plaque, accompanied by different with a degree of itching. Moreover, a rash can occur anywhere on the body, such as the scalp, elbows, knees, waist and trunk.^[5–7]^ Patients with psoriasis are not only greatly affected by their appearance and social life, but also bear heavy psychological pressure. Most patients are accompanied by negative emotions, including anxiety, depression, anger, among others, which lead to a decline in their quality of life.^[8,9]^ Apart from involving body parts such as skin and joints, psoriasis is often associated with some systemic diseases (cardiovascular disease, metabolic disease, inflammatory bowel disease, liver disease, kidney damage, depression, cancer, among others).^[10]^ Besides, recently studies indicate that psoriasis is closely related to various eye diseases (conjunctivitis, scleritis, dry eye).^[11]^

Up to now, the etiology and pathogenesis of PSO have not been clearly elucidated due to its complexity. It is mostly considered to be the result of the interaction of multiple factors (such as infection, genetics, immunity, diet, and environmental factors), among which genetic and environmental factors occupy an important position.^[12]^ The treatment of PSO mainly includes topical therapy, conventional systemic drug therapy, phototherapy, and biological agents according to international guidelines.^[13,14]^ Although it can effectively relieve clinical symptoms and improve the quality of life in the short-term. However, in the long term, it will cause various adverse events, such as abnormal blood lipids, abnormal liver and kidney function, gastrointestinal symptoms, etc. Meanwhile, the price of biologics is so expensive that patients have to bear a heavy financial burden, which is the main reason why most patients are not satisfied with the treatment.^[15]^

Blood-Heat syndrome, as the primary factor in the onset of PSO, forces blood to go into congestion, and blood stasis is formed, which is not only the pathogenesis of repeated and refractory disease, but also is the key to the transition from the progressive period to the stationary period for PSO.^[16]^ Fire needle therapy, as one of the traditional acupuncture and moxibustion therapies of TCM, mainly uses an alcohol lamp to burn the needle body and quickly pierce the local skin lesions, which has the unique effect of promoting blood circulation and removing blood stasis, and warming the meridians.^[17]^ Modern medical research shows that fire needle can improve inflammation, regulate immunity, and central brain function.^[18]^ Therefore, it has been widely used by dermatologist and has a good effect in the treatment of PSO.^[19–21]^ At present, there have been 3 systematic reviews and meta-analyses on the treatment of PSO with fire needle,^[21–23]^ but none of them involve TCM syndrome differentiation as blood stasis syndrome or partial unregistered or lack of evidence quality assessment, among others. It is necessary to systematically evaluate the efficacy and safety of fire needle therapy in the treatment of PSO (blood stasis syndrome), to provide TCM guidance and high level evidence based guidance for the diagnosis and treatment of PSO.

## 2 Methods

### 2.1 Study registration

This system review agreement has been registered on the INPLASY website (registration number: INPLASY202120007).(https://inplasy.com/inplasy-2021-2-0007/). This protocol will be completed according to the Preferred Reporting Items for System review and Meta-analysis Protocol (PRISMA-P) 2015.^[24]^ If there are problems that need to be adjusted during the entire research process, we will make detailed corrections and updates in the final report.

### 2.2 Inclusion criteria

#### 2.2.1 Type of studies

All RCTs of fire needle treatment for PSO (blood stasis syndrome) will be included, regardless of whether blind method is used. Other types of studies such as non-RCTs will be excluded.

#### 2.2.2 Type of participants

The participants in the study must meet both the clinical diagnostic criteria for PSO and the blood stasis syndrome of TCM. There are no restrictions on race, age, sex, lifestyle, education level.

#### 2.2.3 Type of interventions

##### 2.2.3.1 Experimental interventions

Fire needle as the main part of the combined therapy will be included. In addition, single fire needle interventions will also be included.

##### 2.2.3.2 Control interventions

Different types of interventions will be included (eg, single western medicine, traditional chinese herbal, 308-nm excimer laser, other combination therapy, and so on). Other types of interventions such as fire needle will be excluded.

#### 2.2.4 Types of outcome measures

##### 2.2.4.1 Primary outcomes

The primary outcomes include clinical efficacy, psoriasis area and severity index (PASI). The total clinical effective rate is obtained by adding the cure rate and effective rate. PASI score,^[25]^ as an important indicator for evaluating the severity of psoriasis skin manifestations, is stable and reliable.

##### 2.2.4.2 Secondary outcomes

Secondary outcomes were: Itchy (VAS); symptom score according to the evaluation standard of Chinese medicine; dermatological quality of life index (DLQI); adverse events, such as infection, redness, burning, blisters, pigmentation, itching, among others.

### 2.3 Exclusion criteria

Exclusion criteria as follows:

1. Select the latest one among the repeated publications;
2. Women in a special period (pregnancy or lactation);
3. Non blood stasis syndrome;
4. Joint psoriasis, pustular psoriasis, erythroderma psoriasis;
5. Documents whose full text cannot be obtained from various sources;
6. Combined with other serious organic diseases or mental diseases.

### 2.4 Search methods for the identification of studies

#### 2.4.1 Data sources

The following electronic databases will be searched from inception to October 2020: PubMed, Web of Science, Embase, Cochrane Library, China National Knowledge Infrastructure, WangFang Database, Chinese Science Journal Database, and Chinese Biomedical Literature Database. In addition, other documents that meet the requirements will be manually searched, including conference papers, dissertations, among others.

#### 2.4.2 Search strategy

Searching through a combination of medical subject headings and text words, key words include psoriasis, psoriasis vulgaris, plaque psoriasis, BaiBi, fire needle therapy, fire needle, burning red acupuncture, fire acupuncture, randomized controlled trials, controlled clinical trials, clinical trials, trials. Table 1 specifically shows PubMed's search strategy. At the same time, the search method is appropriately modified according to the difference between the Chinese database and the English database.

**Table 1:** Search strategy used in PubMed database.

Order	Search terms
#1	psoriasis[Mesh]
#2	psoriasis vulgaris[title/absteace]
#3	Plaque psoriasis[title/absteace]
#4	psoriases[title/absteace]
#5	psoria[title/absteace]
#6	Baibi[title/absteace]
#7	#1 or #2 or #3 or #4 or #5 or #6
#8	Fire needle therapy[Mesh]
#9	Fire needle[title/absteace]
#10	Burn needle[title/absteace]
#11	Red-hot needle[title/absteace]
#12	Fire-needle[title/absteace]
#13	Fire needling[title/absteace]
#14	Fire Acupuncture[title/absteace]
#15	#8 or #9 or #10 or #11 or #12 or #13 or #14
#16	Randomized controlled trials[title/absteace]
#17	Controlled clinical trial[title/absteace]
#18	Clinical trial[title/absteace]
#19	Tial[title/absteace]
#20	#16 or #17 or #18 or #19
#21	#7 and #15 and #20

### 2.5 Data collection

#### 2.5.1 Studies selection

**Figure 1:**
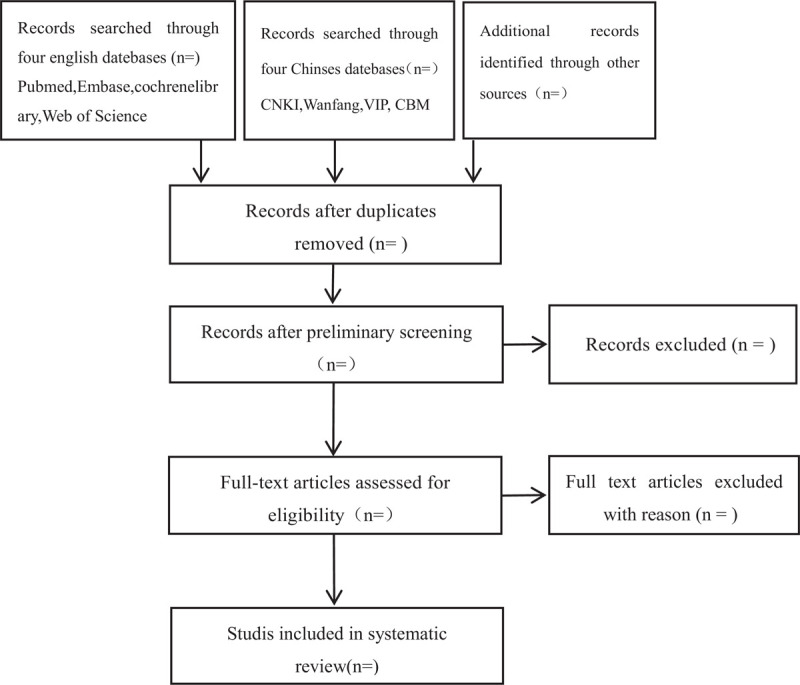
Flow diagram of study selection process.

Based on the above search strategy, researchers will import the research that meet the requirements into noteexpress3.2.0, and then discard the repeated research. Two researchers (JHZ and JY) independently completed the screening work. The screening process is divided into 3 parts: browse the title and abstract of the literature and initially exclude the literature that did not meet the inclusion criteria; re-screen by reading the full literature carefully; check the results and finally discuss whether the literature is included. If 2 researchers have different opinions, we will invite the third reviewer (GH) to make the final result. The literature selection process is shown in Figure 1.

#### 2.5.2 Data extraction and management

The data will be extracted independently by the 2 researchers (JHZ and QH), then cross-check and verify each other. Any disagreement will be resolved by discussion with a third reviewer (CXH). The extracted data mainly include the following information: literature information (first author, publication year, study area), research methods, participant characteristics, sample size, intervention (s), outcome (s), adverse event (s), and other relevant characteristics.

### 2.6 Data analysis

#### 2.6.1 Assessment of risk of bias in included studies

Two researchers (JHZ and PZC) will use the Cochrane risk assessment tool to evaluate the included literature, including the following 7 aspects: random sequence generation, allocation concealment, blinding of participants and researchers and outcome assessors, incomplete outcome data, selective outcome reporting, and other bias. The assessments will be classified into 3 levels: low risk, high risk, and unclear risk. Selecting “yes” indicates that there is a low risk; “no” indicates that there is a high risk; “unclear” indicates that there is a risk of uncertain deviation. Issues are resolved by rechecking the original document and further discussions with the third reviewer (GH).

#### 2.6.2 Measures of treatment effect

When the analysis data are continuous data, results will be reported as mean difference or standard mean difference with 95% confidence interval (95%CI). The dichotomous data will be calculated with the odds ratio with 95% CI.

#### 2.6.3 Dealing with missing data

Missing data will be obtained by contacting the first author or corresponding author of the study by phone or email. If the author cannot be contacted, we will only analyze the existing data and further discuss the potential impact of missing data.

#### 2.6.4 Assessment of heterogeneity

We will detect the heterogeneity between studies by *χ*^2^ test and Higgin *I*^*2*^ test. When *P* > .1 and *I*^*2*^ <50%, there is no obvious heterogeneity and the fixed-effects model will be used; otherwise, the random effects model will be used for merger of the analysis.

#### 2.6.5 Data synthesis

RevMan software (Version5.3, Copenhagen: The Nordic Cochrane Center, The Cochrane Collaboration, 2014)will be applied in this meta-analysis. We will use the fixed effects model or the random effects model according to the results of the heterogeneity test. When there is significant clinical heterogeneity between studies, descriptive analysis will be performed. The forest plots will be used to display the results of this meta-analysis.

#### 2.6.6 Subgroup analysis

If significant heterogeneity is detected in all studies, we will try to conduct a subgroup analysis based on patient characteristics, interventions (including simple fire needles, fire needles combined with different drugs or physical therapies) and treatment time.

#### 2.6.7 Sensitivity analysis

If necessary, sensitivity analysis is performed according to the sample size and methodology of the research to obtain a stable and reliable result.

#### 2.6.8 Assessment of publication bias

When the number of studies in the meta-analysis is large enough (RCTs >10), the funnel plot is used to assess publication bias.

#### 2.6.9 Summary of evidence

Under the systematic review, GRADE method is used to evaluate the evidence of all research results. The level of evidence quality reflects its confidence in the effect estimation. It is mainly divided into 4 levels: high quality, medium quality, low quality, and very low quality.^[26]^

#### 2.6.10 Ethics and dissemination

Ethical review and approval are not required because individual data and privacy of participant will not be involved. The results of this systematic review will be published in peer-reviewed publications.

## 3 Discussion

PSO is mainly characterized by the rapid proliferation of epidermal keratinocytes, which is closely related to a variety of comorbidities.^[10,11]^ In addition, seasonal changes, psychological stress, obesity, diet, tobacco, and alcohol are potential risk factors for recurrence or aggravation of psoriasis.^[27,28]^ Whether it is physical burden or psychological pressure, it brings huge distress to patients and affects the quality of life of patients seriously.^[29]^ Therefore, it is necessary to find an economical, simple, and effective treatment method intervene in PSO.

Fire needle therapy, as an important part of TCM characteristic therapies, has the characteristics of simple operation, low cost and high safety. In recent years, more and more research teams have conducted RCTs of fire needle therapy for PSO and published them publicly.^[30,31]^ Therefore, it is worthy of systematically evaluating them to establish convincing evidence to prove the effectiveness and safety of Fire needle for PSO. However, this systematic review may also have some limitations and insufficiency. First of all, the blind method cannot be realized due to the particularity of the fire needle operation. Second, the overall quality of the RCTs may not be high, which affects the reliability of the research results.

## Author contributions

**Conceptualization:** Jiahua Zou, Gang Huang, Liping Gong.

**Data curation:** Jiahua Zou, Juan Yan, Feiyan Zhang.

**Formal analysis:** Dongbei Huang, Chuxiang Hu, Qiang Huang, Peize Chen.

**Investigation:** Liping Gong, Gang Huang, Jiahua Zou.

**Methodology:** Jiahua Zou.

**Project administration:** Jiahua Zou, Juan Yan.

**Software:** Chuxiang Hu, Juan Yan.

**Supervision:** Liping Gong.

**Writing – original draft:** Gang Huang, Jiahua Zou.

**Writing – review & editing:** Liping Gong.
